# Impact of Hospitalist-Led Care on Glycemic Control Among Hospitalized Adults with Diabetes in Korea

**DOI:** 10.3390/jcm15020406

**Published:** 2026-01-06

**Authors:** Soohyun Lee, Jaewoong Kim, Areum Shin, Sunhee Jo, Chul Sik Kim, Taeyoung Kyong

**Affiliations:** 1 Department of Hospital Medicine, Yongin Severance Hospital, Yonsei University College of Medicine, 363 Dongbaekjukjeon-daero, Giheung-gu, Yongin 16995, Republic of Korea; ishy87@yuhs.ac (S.L.); martins00@yuhs.ac (J.K.); dkfma20427@yuhs.ac (A.S.); xxshjo@yuhs.ac (S.J.); 2 Department of Biomedical Systems Informatics, Yonsei University College of Medicine, Seoul 03722, Republic of Korea; 3 Division of Endocrinology & Metabolism, Department of Internal Medicine, Yongin Severance Hospital, Yonsei University College of Medicine, Yongin 16995, Republic of Korea; ironeat@yuhs.ac

**Keywords:** glycemic control, hospitalist, coefficient of variation

## Abstract

**Background/Objectives**: Hyperglycemia in hospitalized patients is associated with an increased risk of complications, morbidity, mortality, and healthcare costs, regardless of a prior diagnosis of diabetes. The hospitalist system can improve various outcomes, including length of stay, medical costs, patient satisfaction, and mortality rates. However, the effects of hospitalist care on blood glucose control in hospitalized patients remain unclear. This study aimed to assess the specific effects of hospitalist services on blood glucose control in hospitalized patients, with a focus on hyperglycemia management and patient outcomes. **Methods:** This retrospective study reviewed the electronic medical records of patients diagnosed with diabetes at Yonsei Severance Hospital in Yongin, between March 2020 and February 2022. It included adults aged ≥20 years who were hospitalized and had undergone blood glucose measurements during hospitalization. Glycemic control was assessed using hemoglobin A1c, and the blood glucose levels were measured four times daily during hospitalization. Variability was quantified using the coefficient of variation and compared between hospitalist-led and traditional specialty care groups, over a 14-day hospitalization period. **Results:** Despite a higher baseline risk profile, patients receiving hospitalist-led care experienced significantly more stable glycemic variability over time (*p* = 0.002), suggesting better inpatient glucose management than those receiving traditional specialty care. **Conclusions:** Hospitalist-led care was associated with more stable glycemic variability over time in hospitalized patients with diabetes, despite a higher baseline burden of comorbidities and poorer glycemic control at admission.

## 1. Introduction

Diabetes mellitus is a major global health problem and one of the most prevalent chronic diseases in South Korea. According to the Diabetes Fact Sheet in Korea 2024, the prevalence of diabetes among Korean adults aged ≥30 years was approximately 15.5% in 2021–2022, with significant implications for morbidity, mortality, and healthcare costs [1]. The burden of diabetes is further compounded by its association with cardiovascular disease, renal complications, and reduced quality of life.

Hyperglycemia is prevalent in hospitalized patients, poses a significant health challenge, and is associated with a number of negative outcomes.

Hyperglycemia—prevalent in hospitalized patients regardless of diabetes status—is associated with an elevated risk of infection [2,3], fluid and electrolyte imbalances, and impaired wound healing, which is primarily due to osmotic diuresis that leads to reduced oxygenation and perfusion [4]. Furthermore, hyperglycemia plays a crucial role in cardiovascular events [5], either by inducing cardiac myocyte death through apoptosis or by exacerbating cellular injury during ischemic reperfusion.

Uncontrolled hyperglycemia is also associated with diminished neurologic recovery [6], prolonged hospital length of stay (LOS) [7,8,9], escalated healthcare costs [10], and increased in-hospital mortality rates [7,9,11]. Randomized clinical trials involving patients with hyperglycemia have reported the potential benefits of controlled blood glucose levels in mitigating these issues. Additionally, a dedicated glycemia management team can help in effectively managing hyperglycemia in hospitalized patients [12].

The demand for hospitalists in Korea increased following regulatory adjustments to training resident work hours and the shortening of internal medicine and general surgery residencies [13]. This shift promoted the need for a hospitalist system, which was initiated through pilot programs in November 2015 [14] and officially established with a dedicated fee for hospitalist services on 27 November 2020 [15].

The concept of hospitalist has numerous potential advantages over traditional inpatient treatment models [16]. By focusing exclusively on inpatient care, hospitalists can swiftly address acute symptoms or respond to new test results, minimizing delays and enhancing overall patient outcomes [16,17,18]. The hospitalist system has previously demonstrated improvements across various aspects, including the LOS [19,20], re-admission rates [21], emergency department waiting periods [19], medical costs [17], patient satisfaction [22], and mortality rates [23].

Although the positive effects of the hospitalist model on various clinical outcomes are well-established, its influence on blood glucose control in hospitalized patients remains largely unexplored. Consequently, in this retrospective study, we aimed to evaluate the association between hospitalist-led care and glycemic variability in hospitalized adults with diabetes, with a focus on hyperglycemia management and patient outcomes. Unlike previous studies that have primarily focused on efficiency, LOS, or mortality rates, this study specifically examined the impact of the hospitalist model on inpatient glycemic control. It also highlighted a novel and understudied aspect of hospital medicine. Our study offers valuable insights into the benefits of the hospitalist model on inpatient care.

### Hospitalist Model and Context at Yongin Severance Hospital

In the Korean hospital system, hospitalists are full-time inpatient physicians dedicated exclusively to hospital-based care, with no outpatient duties. In contrast, traditional specialty departments provide both outpatient and inpatient services, resulting in differences in workflow and response times for inpatient management. These structural characteristics are essential for interpreting comparisons between hospitalist-led care and traditional specialty care in this study.

In Yongin Severance Hospital, hospitalists are exclusively affiliated with the Department of Hospital Medicine. They primarily manage patients with complex medical conditions, those recently discharged from the intensive care unit, and acutely ill patients admitted through the emergency department. In contrast, physicians in other clinical departments provide both inpatient and outpatient services and do not function as hospitalists. This distinction in scope of practice and training was clarified to avoid confusion regarding the role of hospitalists in Korea. Meanwhile, patients with relatively mild conditions or those admitted for short-term planned procedures, such as chemotherapy or elective interventions, are typically managed by their respective clinical departments. When classifying the clinical departments, family medicine, rehabilitation medicine, dermatology, neurology, and psychiatry were categorized as internal medicine departments. In contrast, urology, obstetrics and gynecology, ophthalmology, otorhinolaryngology, and orthopedics were classified as surgical departments. Ultimately, 28 clinical departments were included in this study.

## 2. Materials and Methods

### 2.1. Study Design

**Figure 1:**
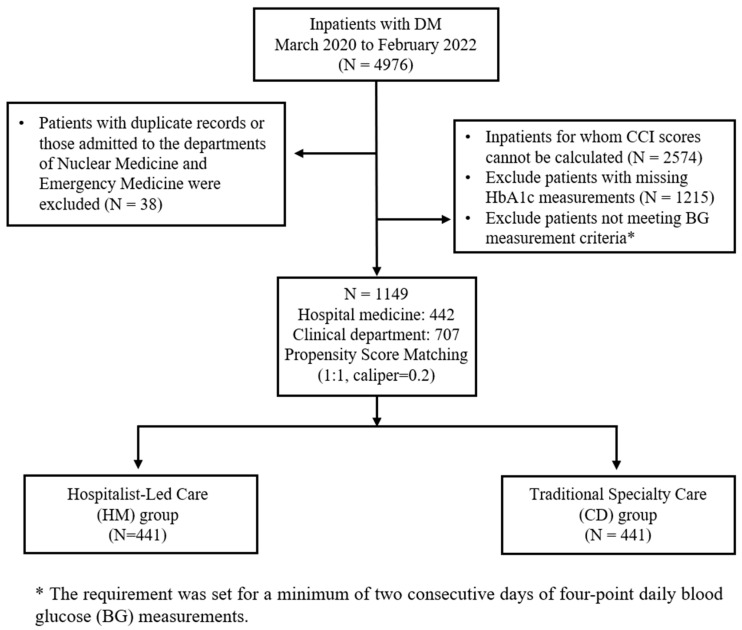
Study population flowchart showing patient selection and propensity score matching processes.

This retrospective study analyzed the electronic medical records of patients with diabetes, admitted to the Yongin Severance Hospital between March 2020 and February 2022. All types of diabetes (type 1, type 2, and other specified forms) were included, identified using the International Classification of Diseases, Tenth Revision (ICD-10) codes E11, E13, E14, G59, G63, and H36. It compared inpatient glycemic control between the patients in the Department of Hospital Medicine (Hospitalist-Led Care, HM group) and other clinical departments (Traditional Specialty Care, CD group). The study population included adult patients aged ≥20 years who underwent four-point daily blood glucose measurements for at least two consecutive days during hospitalization. Patients admitted to the intensive care unit, those aged <20 years, and those admitted to the emergency and nuclear medicine departments were excluded (Figure 1). To reflect the patient’s baseline severity and prior glycemic control status, the first day of hospitalization was included even if fewer than four measurements were recorded.

In addition, inpatient diabetes care at Yongin Severance Hospital is guided by regularly updated Inpatient Diabetes Management Guidelines. These institutional protocols, developed collaboratively by hospitalists, endocrinologists, and clinical pharmacists, standardize blood glucose monitoring frequency, insulin titration, perioperative glucose control, and the management of hyperglycemia and hypoglycemia across all departments.

Crucially, all departments, including both Hospitalist-Led Care and Traditional Specialty Care, followed the same protocol for blood glucose measurement frequency (four times daily, typically before meals and at bedtime) and management targets. Incorporating these guidelines ensures consistency in inpatient glycemic management and provides important context for interpreting the outcomes of this study, noting that while protocols were identical, differences may exist in adherence and rapidity of intervention.

### 2.2. Inpatient Glycemic Management Protocol

Glycemic control was managed according to the institutional Inpatient Diabetes Management Guidelines. The primary strategy employed a basal-prandial insulin regimen, consisting of long-acting insulin (basal) and rapid-acting insulin analogues (prandial) administered in a 50:50 ratio of the total daily dose. Long-acting insulins included Tresiba^^®^^ (insulin degludec; Novo Nordisk A/S, Copenhagen, Denmark) and Toujeo^®^ (insulin glargine; Sanofi-Aventis, Paris, France). Rapid-acting insulins included Fiasp^®^ (insulin aspart; Novo Nordisk A/S, Copenhagen, Denmark), NovoRapid^®^ (insulin aspart; Novo Nordisk A/S, Copenhagen, Denmark; also manufactured in Chartres, France), and Apidra^®^ (insulin glulisine; Sanofi-Aventis Deutschland GmbH, Frankfurt am Main, Germany). To enhance precision and safety, insulin doses were determined and adjusted using an Automatic Insulin Dosing (AID) system, an in-house developed software integrated into the electronic medical record at Yongin Severance Hospital (Yongin, Republic of Korea). As this is a proprietary institutional program, no commercial version number is applicable. The AID system recommends individualized doses based on real-time glucose levels and meal intake. Instead of a conventional sliding scale, a correction insulin protocol was applied to address pre-prandial hyperglycemia. Detailed algorithms for dose adjustment and the AID system framework are provided in Supplementary Table S1 and Supplementary Figure S1.

### 2.3. Glycemic Variability

To assess glycemic variability, only patients who underwent four-point blood glucose measurements per day, on consecutive days during hospitalization, were included in the analysis. To ensure consistency, we included the first day of hospitalization even if fewer than four measurements were recorded, as admission-day glucose values provide clinically meaningful information regarding patient severity and prior glycemic control. Measurements were typically obtained before each meal and at bedtime. No imputation was performed; only complete and valid glucose measurements were used for all analyses. Glycemic variability was quantified using the coefficient of variation (CV), calculated by dividing the standard deviation of daily blood glucose levels by the corresponding mean glucose level and expressed as a percentage. This enabled standardized comparisons across individuals with varying mean glucose levels. Hypoglycemia frequency was defined as the number of blood glucose readings < 70 mg/dL among four-point blood glucose measurements per day, on consecutive days during hospitalization.

### 2.4. Statistical Analysis

Continuous variables are expressed as medians and interquartile ranges, whereas categorical variables are expressed as frequencies and proportions. Normality tests were conducted before comparing group differences, and parametric and non-parametric methods were applied. Group differences in continuous variables were analyzed using the *t*-test or the Wilcoxon signed-rank test. In contrast, differences in categorical variables were assessed using the chi-squared test. To minimize selection bias and balance the key baseline characteristics, Greedy 1:1 propensity score matching (PSM) was performed using a caliper of 0.2. The variables used for matching were LOS, Charlson Comorbidity Index (CCI), and surgery status. The quality of matching was evaluated using standardized mean differences (SMDs), with an SMD < 0.1 indicating adequate balance. Patients with complete data were included in the analysis, and those with missing values for any of the study variables were excluded. To analyze the factors associated with glycemic variability, while accounting for repeated measurements during hospitalization, a generalized estimating equations (GEE) model was applied. The frequency of hypoglycemia was analyzed using negative binomial regression. All statistical analyses were conducted using the SAS software (version 9.4; SAS Institute Inc., Cary, NC, USA) and R software (version 4.3.1; R Foundation for Statistical Computing, Vienna, Austria). Statistical significance was set at *p* < 0.05.

### 2.5. Ethics Statement

The study protocol was approved by the Institutional Review Board (IRB) of Yonsei Yong-in Severance Hospital (IRB number: 2022-0047-001). The requirement for written informed consent from the participants was waived.

## 3. Results

### 3.1. Baseline Characteristics

In total, 1149 patients were included in the analysis before PSM, with 442 and 707 patients in the HM and CD groups, respectively. Before PSM, statistically significant differences in LOS (*p* = 0.042), CCI score (*p* = 0.014), and operative procedure status (*p* < 0.001) were observed between the two groups. Subsequently, these variables were selected for the PSM. After 1:1 nearest-neighbor matching using a caliper of 0.2, 441 patients were included in each group. Following matching, no statistically significant differences in age at diagnosis, LOS, CCI score, body mass index (BMI), operative status, steroid use, or hemoglobin A1c (HbA1c) levels were observed between the two groups, indicating adequate balancing of the baseline characteristics (Table 1). Although patients in the HM group had slightly higher HbA1c levels than those in the CD group, the differences were not statistically significant, both before and after matching (Table 1). Furthermore, no significant intergroup differences were observed before or after matching for most comorbidities, including hypertension, hyperlipidemia, malignancy, dementia, cerebrovascular accidents, chronic obstructive pulmonary disease, and chronic kidney disease. However, congestive heart failure remained significantly different between the groups after matching and was therefore included as a covariate in the multivariable analysis using the GEE model. Additionally, a statistically significant difference for congestive heart failure was observed between the groups after matching (*p* = 0.046), although it was not included in the initial matching variables.

### 3.2. Comparison of Glycemic Variability Between Groups over Time

**Figure 2:**
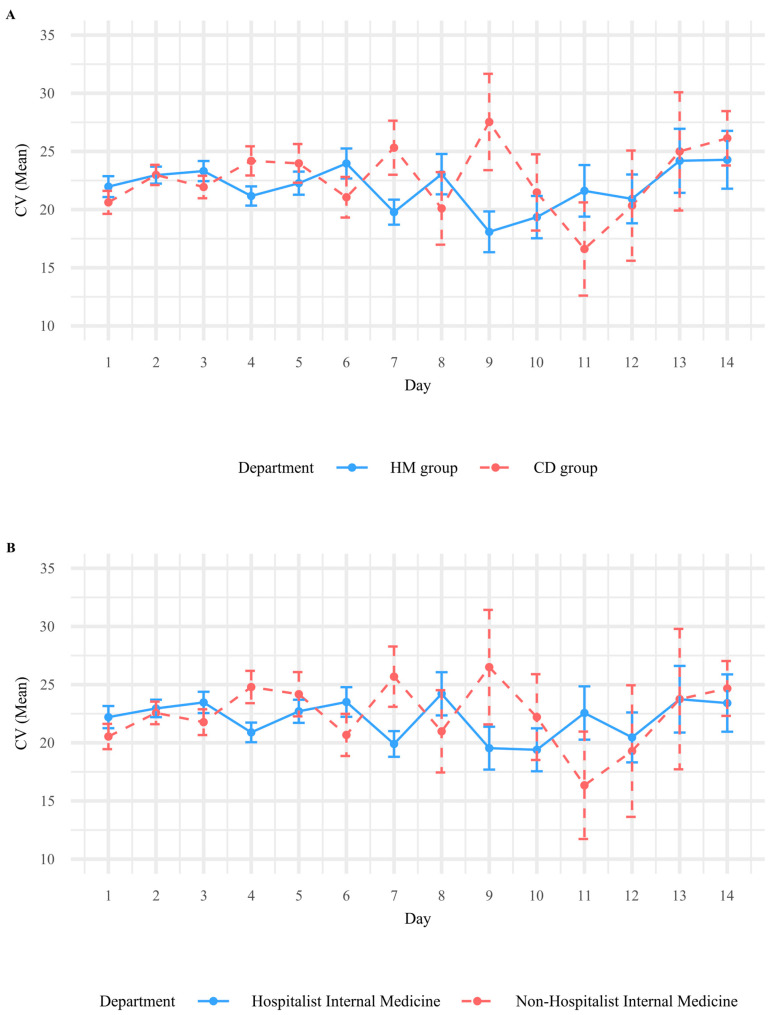
(**A**) Comparison between Hospitalist-led Care (HM) and Traditional Specialty Care (CD). In Korea, hospitalists are full-time inpatient physicians dedicated solely to hospital-based care, whereas traditional specialty departments manage both outpatient and inpatient services. (**B**) Subgroup comparison within internal medicine services: Hospitalist Internal Medicine vs. Non-Hospitalist Internal Medicine. Hospitalist Internal Medicine refers to internal medicine patients managed under the Department of Hospital Medicine, while Non-Hospitalist Internal Medicine refers to internal medicine patients admitted under other specialty departments.

**Figure 3:**
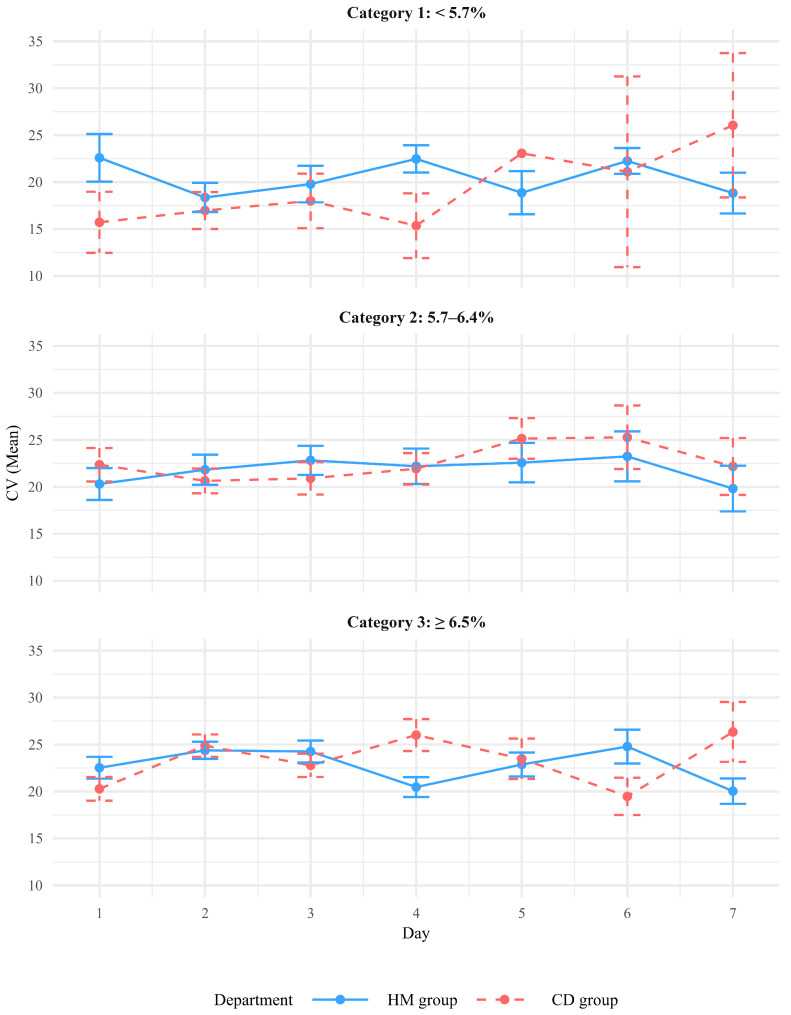
Coefficient of variation (CV) over 7 days by HbA1c category.

Figure 2A illustrates the changes in glycemic variability, represented by CV, over a 14-day hospitalization period in the HM and CD groups. Overall, the CD group showed slightly greater day-to-day fluctuations in CV compared with the HM group. In the GEE analysis (Table 2), the time (in days) was significantly associated with increasing glycemic variability (estimate = 0.484, *p* = 0.046), whereas the quadratic term for time (Day^2^) was negatively associated with it (estimate = −0.016, *p* = 0.008), suggesting a nonlinear trajectory. The HM group further exhibited a higher average CV than the CD group; however, the difference was not statistically significant (estimate = 1.383, *p* = 0.111). A significant interaction was observed between time and department (estimate for Day × HM = −0.394, *p* = 0.002), indicating that the trajectory of glycemic variability over time differed between the HM and CD groups. Specifically, patients in the HM group demonstrated a more gradual increase in glycemic variability over time (Table 2). The GEE model, adjusted for HbA1c category (category 1: <5.7%; category 2: 5.7–6.4%; and category 3: ≥6.5%; Table 3, Figure 3), revealed a consistent pattern, confirming the difference in glycemic variability trajectories between the HM and CD groups. Specifically, the interaction between time and the HM group was statistically significant (estimate = −0.350, *p* = 0.007), indicating that patients in the HM group experienced more stable glycemic variability over time. Additionally, patients in the CD group with higher HbA1c levels (categories 2 and 3) showed significantly greater glycemic variability than those with lower HbA1c levels (estimate = 4.057, *p* = 0.023 for category 2 and estimate = 4.937, *p* = 0.006 for category 3) (Table 3). This suggested that poor baseline glycemic control may contribute to increased glycemic variability during hospitalization.

Abbreviations: HM group, Hospitalist-Led Care; CD group, Traditional Specialty Care.

### 3.3. Comparison of Glycemic Variability Between Hospital and Clinical Internal Medicine Groups

A subgroup analysis was performed, including only patients admitted to internal medicine departments, further comparing those admitted to the departments of Hospital Internal Medicine (HIM group) and Clinical Internal Medicine (CIM group). The patients in the CIM group exhibited greater variability in glycemic control over time, with more pronounced fluctuations in CV than those in the HIM group (Figure 2B). In the GEE analysis (Supplementary Table S2), although the linear effect of time was marginally significant (estimate = 0.509, *p* = 0.063), the quadratic time term (Day^2^) remained significantly negative (estimate = −0.017, *p* = 0.017), indicating a nonlinear trend in glycemic variability. The HIM group exhibited a higher average CV; however, this difference was not statistically significant (estimate = 1.761, *p* = 0.063). The interaction between time and the HIM group remained statistically significant (estimate = −0.436, *p* = 0.001), suggesting that patients in the HIM group experienced a more stable trend in glycemic variability over time than those in the CIM group. The results were consistent in the model adjusted for HbA1c categories (Supplementary Table S3), in which the time-by-department interaction (estimate = −0.378, *p* = 0.006) remained significant. Additionally, patients in the CIM group with higher HbA1c categories (categories 2 and 3) showed significantly higher CV (estimate = 4.451, *p* = 0.046 for category 2 and estimate = 5.099, *p* = 0.005 for category 3), highlighting the impact of poor glycemic control on glycemic variability during hospitalization.

The frequency of hypoglycemia events tended to be lower in the HIM group compared with the CIM group (estimate = −0.099, *p* = 0.620), although this difference was not statistically significant (Supplementary Table S4).

## 4. Discussion

Numerous studies have demonstrated that hospitalists—physicians specializing in inpatient care—can improve the quality of care and patient safety [16,17,18], and reduce healthcare costs [17] by shortening LOS [19,20]. The prevalence of diabetes continues to rise alongside the increasing life expectancy and greater utilization of healthcare services [24]. In this context, inpatient glycemic control has been recognized as a critical factor influencing the clinical outcomes in hospitalized patients.

In South Korea, several studies have demonstrated the beneficial effects of the hospitalist system on patient care since its introduction in 2015. Consequently, hospitalist programs have been increasingly adopted, particularly in tertiary care hospitals. Various indices, including glycemic variability, mean of daily differences, and HbA1c, have been proposed to evaluate the quality of inpatient glycemic management. Increased glycemic variability is globally recognized as being independently associated with a higher risk of adverse outcomes, including nosocomial infections, delayed wound healing, and cardiovascular complications in hospitalized patients. Previous studies, conducted in Korea, have shown that increased glycemic variability is independently associated with a higher risk of nosocomial infections, delayed wound healing [25], and cardiovascular complications [26,27,28] in hospitalized patients. Thus, stabilizing glycemic fluctuations during hospitalization is not only a matter of numerical control but a determinant of broader clinical outcomes.

In the present study, we quantified glycemic variability using CV and assessed glycemic control using HbA1c levels. The CV reflects the degree of fluctuation in blood glucose relative to the daily average. A higher CV indicates greater variability, suggesting that blood glucose levels fluctuate widely between hyperglycemia and hypoglycemia throughout the day rather than remaining stable [26,29,30,31,32,33]. This variability may have an even greater clinical significance than elevated mean glucose levels. Rapid glycemic fluctuations are associated with adverse outcomes such as cardiovascular disease, neuropathy, and microvascular complications. In an inpatient setting, such instability can further compromise patient conditions and complicate clinical management.

We assessed inpatient glycemic variability using CV among patients with consistent, scheduled glucose monitoring. This standardized approach enabled meaningful comparison of glucose fluctuations across individuals. Notably, our findings align with and expand on prior findings on structured inpatient diabetes care. Toyoshima et al. demonstrated that hospitalist-led, protocol-driven insulin management using a mobile application maintained 71% of glucose values within the target range [34]. In addition, Wexler et al. showed that a computerized insulin order set improved glycemic control without increasing hypoglycemia [35]. Schnipper et al. further highlighted the benefits of combining insulin protocols with provider education, leading to greater use of basal–bolus regimens and better glucose outcomes [36].

However, these studies primarily focused on mean glucose levels or protocol adherence. In contrast, our study uniquely evaluated glycemic variability—a clinically meaningful and underexplored marker of inpatient glycemic control—within a hospitalist-led care model. To our knowledge, this is the first such study conducted in Korea, and it contributes to the global evidence base by adding new insights into the value of hospitalist-driven interventions in optimizing diabetes care during hospitalization.

The initially higher mean CV observed in the HM group than in the CD group is likely attributable to the greater proportion of patients with higher CCI scores and elevated HbA1c levels in the HM group (Supplementary Figures S2 and S3). This suggests that patients admitted under hospitalist care often have more severe comorbid conditions and poorer pre-admission glycemic control.

Nevertheless, the findings that glycemic variability decreased throughout hospitalization and that the HM group had a shorter average LOS suggest the need for more intensive and effective inpatient management. These results imply that hospitalist-led care may offer more focused and coordinated glucose management than traditional specialty care, even in populations with higher baseline risk. Although the difference was not statistically significant, a trend toward fewer hypoglycemic events was observed in the HIM group compared with the NHIM group, suggesting a possible benefit in glycemic safety. Unlike traditional specialty services, hospitalist-led care ensures continuous ward presence, enabling a timely response to glucose fluctuations and better coordination with other disciplines.

An additional contextual factor is the standardized approach to inpatient diabetes care at our institution. Although all departments adhere to the Inpatient Diabetes Management Guidelines, hospitalists tend to implement them with greater consistency and timeliness, supported by their continuous ward presence and close collaboration with nursing and multidisciplinary teams. This structured care model may have contributed to the more stable glycemic trajectories observed in the hospitalist group and enhances the generalizability of our findings to other hospitalist models worldwide.

To ensure a clinically homogeneous population, the subgroup analysis was restricted to patients admitted to internal medicine departments. Surgical patients were excluded because perioperative glycemic fluctuations—driven by surgical stress, anesthesia, fasting protocols, and short elective admissions—produce variability patterns that differ markedly from those in medical inpatients [37,38,39,40]. Limiting the analysis to internal medicine services, therefore, allowed for a more accurate and meaningful comparison between hospitalist-led and non-hospitalist medical care.

This study has certain limitations that should be considered when interpreting the findings. In addition, several clinically relevant variables could not be captured in the dataset and, therefore, were not adjusted for in our analyses. The duration of diabetes was unavailable, although longer disease duration is associated with progressive β-cell dysfunction and greater glycemic lability, potentially contributing to residual confounding. Medication adherence during hospitalization—including the timing, completeness, and protocol adherence of insulin administration—also could not be evaluated and may have varied across departments, potentially influencing day-to-day glucose variability. Furthermore, acute illness severity and stress-related factors such as infection, postoperative inflammation, high-dose steroid exposure, and fluctuations in nutritional intake were not included in the structured dataset despite their substantial impact on inpatient glycemic fluctuations. These unmeasured confounders should be considered when interpreting the observed differences in glycemic variability between care models. Beyond these issues, the retrospective design inherently poses the risk of selection bias and unmeasured confounding factors. Although PSM was applied to reduce baseline imbalances between groups, residual confounders related to clinical status or inpatient management practices may remain. Additionally, only patients with four-point daily glucose monitoring on consecutive days were included, which, while necessary for temporal consistency, may have excluded patients with shorter stays or less intensive monitoring, potentially limiting the generalizability of the results to all hospitalized patients with diabetes. The classification of departments into medical and surgical specialties was simplified and may not fully reflect the clinical complexity or interdisciplinary nature of care in some cases. Finally, because this study was conducted at a single tertiary hospital in South Korea, differences in institutional protocols, staffing models, or patient demographics in other settings may limit the generalizability of these findings.

In summary, this study demonstrates that hospitalist-led care is associated with more stable glycemic variability over time in hospitalized patients with diabetes, despite a higher baseline burden of comorbidities and poorer glycemic control at admission. Although the initial glycemic variability was higher in the HM group, the rate of increase in variability during hospitalization was significantly attenuated compared with that in patients in the CD group. These findings support the potential of hospitalist-led models to deliver more effective and consistent inpatient glycemic management, which may translate into better clinical outcomes, enhanced patient safety, and reduced hospital resource utilization. Further prospective and multicenter studies are warranted to validate these findings and explore the mechanisms underlying the benefits of hospitalist care in diabetes management.

## Figures and Tables

**Table 1:** Baseline characteristics of patients before and after PSM.

	Before PSM			After PSM		
Variable	HM Group	CD Group	*p*	HM Group	CD Group	*p*
	N = 442	N = 707		N = 441	N = 441	
Sex, male	243 (55.0)	386 (54.6)	0.900	242 (54.9)	232 (52.6)	0.500
Age at diagnosis	73.0 (63–79)	71.0 (61–79)	0.208	73.0 (63–79)	72.0 (63–80)	0.814
Length of stay	8 (5–15)	8 (4–15)	0.042	8 (5–15)	8 (5–13)	0.158
Charlson Comorbidity Index	2 (1–3)	2 (1–3)	0.014	2.0 (1–3)	2.0 (1–3)	0.956
Body mass index	24.3 (21.8–27.5)	24.8 (22.3–27.0)	0.558	24.4 (21.8–27.5)	24.5 (22.1–26.9)	0.793
Operative procedure status			<0.001			>0.999
Yes	50 (11.3)	158 (22.4)		49 (11.1)	49 (11.1)	
No	392 (88.7)	549 (77.6)		392 (88.9)	392 (88.9)	
Steroid status			0.522			0.937
Use	106 (24.0)	158 (22.4)		105 (23.8)	104 (23.6)	
None	336 (76.0)	549 (77.6)		336 (76.2)	337 (76.4)	
HbA1c	6.6 (6.0–7.6)	6.5 (5.9–7.4)	0.213	6.6 (6.0–7.6)	6.5 (6.0–7.5)	0.467
HbA1c level			0.463			0.445
<5.7%	71 (16.1)	110 (15.6)		71 (16.1)	65 (14.7)	
5.7–6.4%	123 (27.8)	221 (31.3)		122 (27.7)	139 (31.5)	
≥6.5%	248 (56.1)	376 (53.1)		248 (56.2)	237 (53.7)	
Comorbidity						
Hypertension	223 (50.5)	341 (48.2)	0.464	222 (50.3)	226 (51.3)	0.788
Congestive heart failure	71 (16.1)	138 (19.5)	0.140	70 (15.9)	93 (21.1)	0.046
Hyperlipidemia	59 (13.4)	73 (10.3)	0.118	59 (13.4)	49 (11.1)	0.304
Malignancy	72 (16.3)	102 (14.4)	0.392	72 (16.3)	67 (15.2)	0.644
Dementia	17 (3.9)	23 (3.3)	0.594	17 (3.9)	17 (3.9)	>0.999
Cerebrovascular accident	36 (8.1)	46 (6.5)	0.294	36 (8.2)	29 (6.6)	0.367
Chronic obstructive pulmonary disease	27 (6.1)	30 (4.2)	0.157	27 (6.1)	25 (5.7)	0.775
Chronic kidney disease	99 (22.4)	141 (19.9)	0.319	98 (22.2)	116 (26.3)	0.157

Abbreviations: PSM, propensity score matching; HM group, Hospitalist-Led Care; CD group, Traditional Specialty Care; HbA1c, hemoglobin A1c.

**Table 2:** Results of generalized estimating equations model explaining variation over time: comparison between HM and CD groups.

Parameter	Reference	Estimate (95% CI)	SE	Z	*p*
Intercept	-	20.820 (19.201 to 22.438)	0.826	25.21	<0.001
Day	-	0.484 (0.009 to 0.960)	0.242	2.00	0.046
Day^2^	-	−0.016 (−0.028 to −0.004)	0.006	−2.67	0.008
Congestive heart failure (yes)	None	0.892 (−0.946 to 2.729)	0.937	0.95	0.342
HM group	CD group	1.383 (−0.319 to 3.084)	0.868	1.59	0.111
Day × HM group	Day × CD group	−0.394 (−0.642 to −0.145)	0.127	−3.10	0.002

Abbreviations: HM group, Hospitalist-Led Care; CD group, Traditional Specialty Care; CI, confidence interval; SE, standard error.

**Table 3:** Results of the generalized estimating equations model, including HbA1c categories: comparison between HM and CD groups.

Parameter	Reference	Estimate (95% CI)	SE	Z	*p*
Intercept	-	16.509 (13.173 to 19.844)	1.702	9.70	<0.001
Day	-	0.483 (0.010 to 0.955)	0.241	2.00	0.045
Day^2^	-	−0.017 (−0.029 to −0.005)	0.006	−2.71	0.007
Congestive heart failure (yes)	None	0.871 (−0.963 to 2.704)	0.936	0.93	0.352
HM group	CD group	4.030 (0.204 to 7.856)	1.952	2.06	0.039
Day × department (HM group)	CD group	−0.350 (−0.602 to −0.098)	0.129	−2.72	0.007
Department × HbA1c (HM group, 5.7–6.4%)	<5.7%	0.869 (−1.739 to 3.477)	1.331	0.65	0.514
Department × HbA1c (HM group, ≥6.5%)	<5.7%	2.194 (−0.247 to 4.635)	1.246	1.76	0.078
Department × HbA1c (CD group, 5.7–6.4%)	<5.7%	4.057 (0.559 to 7.555)	1.785	2.27	0.023
Department × HbA1c (CD group, ≥6.5%)	<5.7%	4.937 (1.429 to 8.446)	1.790	2.76	0.006

Abbreviations: HM group, Hospitalist-Led Care; CD group, Traditional Specialty Care; HbA1c, hemoglobin A1c; CI, confidence interval; SE, standard error.

## Data Availability

The datasets generated during and/or analyzed during the current study are available from the corresponding author upon reasonable request.

## Supplementary Materials

The following supporting information can be downloaded at https://www.mdpi.com/article/10.3390/jcm15020406/s1, Figure S1: Conceptual framework of the Automatic Insulin Dosing(AID) system implemented at Yongin Severance Hospital, illustrating the data flow from patient EMR data to standardized insulin dose recommendation; Figure S2: Boxplot of first-day glucose levels by department; Figure S3: Boxplot of hemoglobin A1c levels by department; Table S1: Insulin dose adjustment and correction protocol; Table S2: Results of the generalized estimating equations model explaining variation over time: comparison between hospital and clinical internal medicine group.; Table S3: Generalized estimating equations parameter estimates including HbA1c categories: comparison between hospital and clinical internal medicine groups; Table S4: Negative binomial regression model estimates for differences in hypoglycemic event rates between hospital medicine and clinical department groups.
