# Supplementary material for: Impact of Hospitalist-Led Care on Glycemic Control Among Hospitalized Adults with Diabetes in Korea
Source: J Clin Med. 2026 Jan 6;15(2):406. doi: 10.3390/jcm15020406 (PMC12841906; doi:10.3390/jcm15020406)
Supplement: Supplementary file 1 [file jcm-15-00406-s001.zip › jcm-3945321-supplementary.pdf]

Supplementary Table S1. Insulin Dose Adjustment and Correction Protocol

Category	Glycemic Target / Threshold	Action & Dosage Adjustment
Glycemic Target	Pre-prandial: 100–140 mg/dL<br>Random/Post-prandial: < 180 mg/dL	Maintain current dose if within target range.
Basal Insulin Adjustment	Fasting Blood Glucose (FBG) > 140 mg/dL	Increase basal insulin by 10–20% or 2–4 units every 2–3 days.
Prandial Insulin Adjustment	Pre-meal (next meal) > 140 mg/dL	Increase the preceding meal's prandial dose by 10% or 1–2 units.
	141–180 mg/dL	Add 1 unit of rapid-acting insulin to the scheduled dose.
Correction Insulin (Sliding Scale)	181–220 mg/dL	Add 2 units of rapid-acting insulin to the scheduled dose.
	> 220 mg/dL	Add 3–4 units and consider notifying the physician for additional evaluation.
Hypoglycemia Management	< 70 mg/dL	Reduce the dose by 20% or 2–4 units; follow the 15/15 rule (15g carbs, recheck in 15 min).

Supplementary Table S2. Results of the generalized estimating equations model explaining variation over time: comparison between hospital and clinical internal medicine groups

**Parameter**	**Reference**	**Estimate (95% CI)**	**SE**	**Z**	***P***
Intercept	-	20.659 (18.862 to 22.456)	0.917	22.53	<0.001
Day	-	0.509 (-0.027 to 1.044)	0.273	1.86	0.063
Day^2	-	-0.017 (-0.030 to -0.003)	0.007	-2.40	0.017
Congestive heart failure (yes)	None	0.633 (-1.350 to 2.615)	1.011	0.63	0.532
Hospital internal medicine	Non-Hospital internal medicine	1.761 (-0.098 to 3.621)	0.949	1.86	0.063
Day × Hospital internal medicine	Day × Non-Hospital internal medicine	-0.436 (-0.700 to -0.171)	0.135	-3.23	0.001

Abbreviations: CI, confidence interval; SE, standard error

[a]"Hospital internal medicine" refers to patients admitted to internal medicine services under the Department of Hospital Medicine. [b]"Clinical internal" refers to patients admitted to internal medicine services under other clinical departments.

Supplementary Table S3. Generalized estimating equations parameter estimates including HbA1c categories: comparison between hospital and clinical internal medicine groups

**Parameter**	**Reference**	**Estimate (95% CI)**	**SE**	**Z**	***P***
Intercept	-	15.613 (11.485 to 19.740)	2.106	7.41	<0.001
Day	-	0.488 (-0.041 to 1.016)	0.270	1.81	0.070
Day^2	-	-0.017 (-0.031 to -0.003)	0.007	-2.44	0.015
Congestive heart failure (yes)	None	0.622 (-1.317 to 2.560)	0.989	0.63	0.530
Hospital internal medicine	Non-Hospital internal medicine	5.286 (0.716 to 9.856)	2.332	2.27	0.023
Day × department (hospital internal medicine)	Non-Hospital internal medicine	-0.378 (-0.648 to -0.108)	0.138	-2.74	0.006
Department × HbA1c (hospital internal medicine, 5.7–6.4%)	< 5.7%	0.408 (-2.261 to 3.078)	1.362	0.30	0.764
Department × HbA1c (hospital internal medicine, ≥ 6.5%)	< 5.7%	2.131 (-0.375 to 4.637)	1.279	1.67	0.096
Department × HbA1c (Non-Hospital internal medicine , 5.7–6.4%)	< 5.7%	4.451 (0.078 to 8.823)	2.231	1.99	0.046
Department × HbA1c (Non-Hospital internal medicine , ≥ 6.5%)	< 5.7%	5.099 (1.537 to 10.300)	1.804	2.65	0.005

Abbreviations: HM group, Hospitalist-Led Care; CD group, Traditional Specialty Care; CI, confidence interval; SE, standard error

[a]"Hospital internal medicine" refers to patients admitted to internal medicine services under the Department of Hospital Medicine. [b]"Clinical internal" refers to patients admitted to internal medicine services under other clinical departments.

Supplementary Table S4. Negative binomial regression model estimates for differences in hypoglycemic event rates between hospital medicine and clinical department groups

**Parameter**	**Reference**	**Estimate (95% CI)**	**SE**	**Wald Chi-Square**	***P***
Intercept	-	-3.147 (-3.428 to -2.865)	0.144	480.09	<0.001
HM group	CD group	-0.099 (-0.492 to 0.293)	0.200	0.25	0.620
Dispersion	-	4.931 (3.793 to 6.410)	0.660		

Abbreviations: HM group, Hospitalist-Led Care; CD group, Traditional Specialty Care; CI, confidence interval; SE, standard error

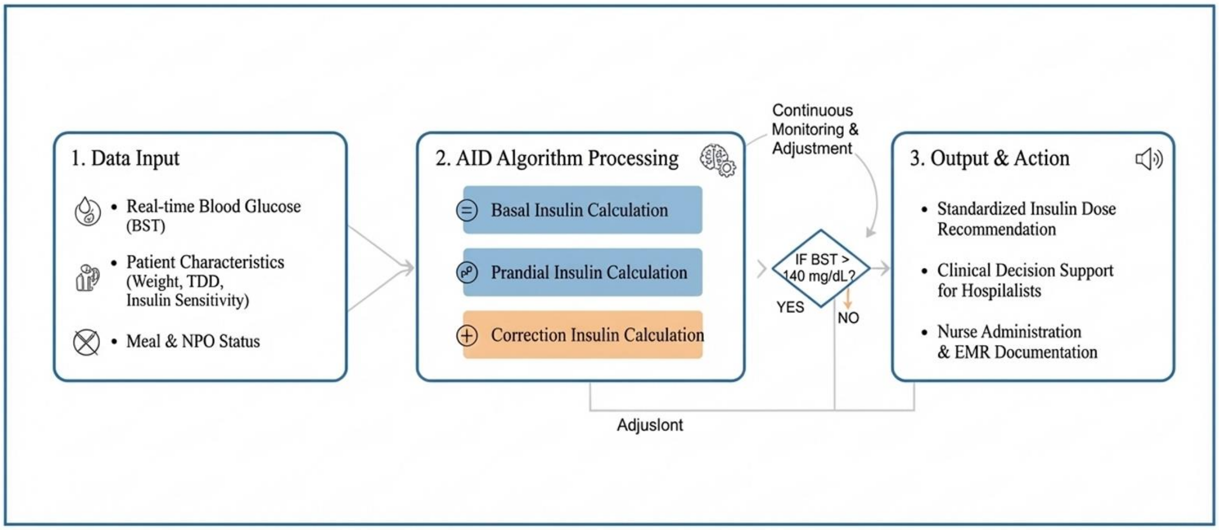

Figure S1: Conceptual framework of the Automatic Insulin Dosing (AID) system implemented at Yongin Severance Hospital, illustrating the data flow from patient EMR data to standardized insulin dose recommendation

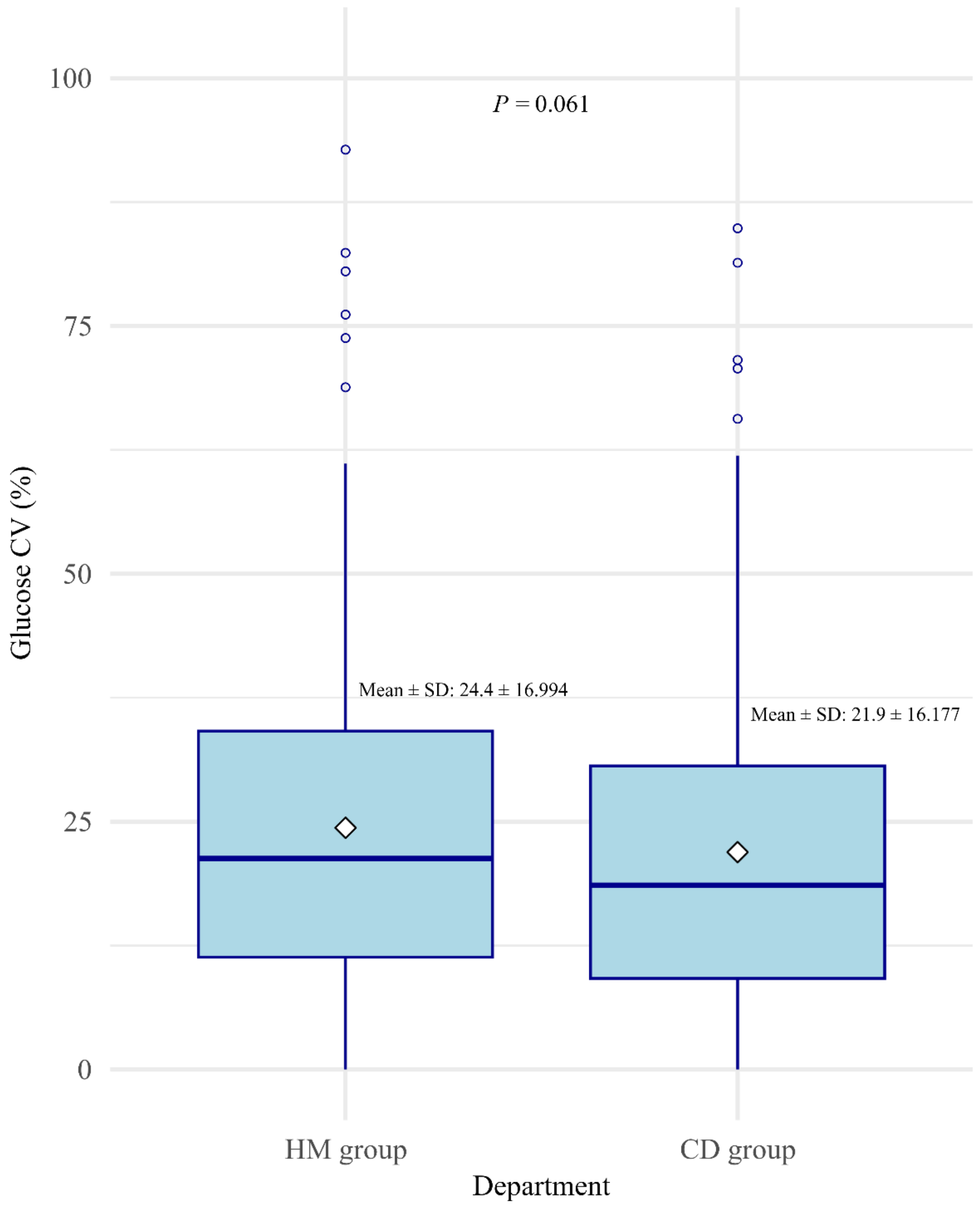

Figure S2: Boxplot of first-day glucose levels by department

Abbreviations: HM group, Hospitalist-Led Care; CD group, Traditional Specialty Care

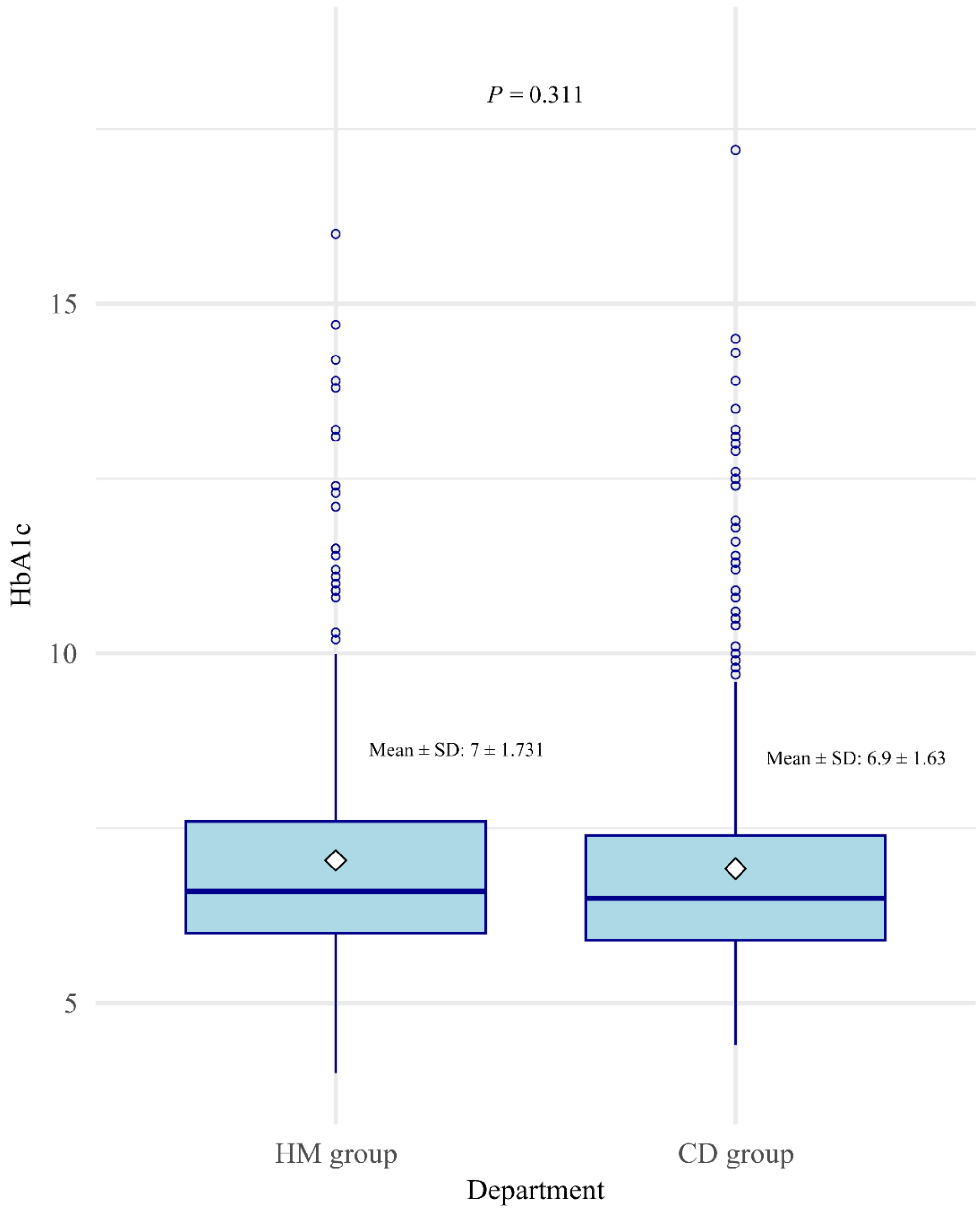

Figure S3: Boxplot of hemoglobin A1c levels by department

Abbreviations: HM group, Hospitalist-Led Care; CD group, Traditional Specialty Care
